# A comparative analysis of agronomic water‐use efficiency and its proxy measures as derived from key morpho‐physiological and supportive quantitative genetics attributes of perennial ryegrass under imposed drought

**DOI:** 10.1002/pei3.10123

**Published:** 2023-08-30

**Authors:** L. V. Y. Weerarathne, Z. Jahufer, R. Schäufele, I. Lopez, C. Matthew

**Affiliations:** ^1^ Department of Crop Science, Faculty of Agriculture University of Peradeniya Peradeniya Sri Lanka; ^2^ School of Agriculture and Environment, College of Sciences Massey University Palmerston North New Zealand; ^3^ School of Agriculture and Food Sciences, Faculty of Science The University of Queensland Brisbane Queensland Australia; ^4^ Crop Physiology, School of Life Sciences Technical University of Munich Freising Germany; ^5^ College of Pastoral Agriculture Science and Technology Lanzhou University Lanzhou China

**Keywords:** deep rooting, drought tolerance, *Lolium perenne* L., osmotic potential, quantitative genetics, water‐use efficiency

## Abstract

Water‐use efficiency (WUE) is an under‐researched but very important drought tolerance trait in forage breeding. This research estimated quantitative genetic parameters of morpho‐physiological traits linked to agronomic water‐use efficiency (WUE_A_) and its proxy measures based on δ^13^C (WUE_i_) or gas exchange (evapotranspiration, WUE_AET_, or stomatal conductance WUE_ASC_) of genotypes from half‐sib families of *Lolium perenne* L. (PRG) in a simulated summer drought cycle. Principal component analysis (PCA) of trait data distinguished a group of PRG genotypes where high WUE_A_ and dry matter yield was associated with deep rooting, leaf hydration at more negative leaf osmotic and water potential, and reduced soil moisture depletion. Plants with this trait association sustained net assimilation and postdefoliation regrowth in drought. However, WUEi, WUE_ASC_, and WUE_AET_ were poorly correlated with most traits of interest at *p* < .05. Another PCA revealed a weak association between WUE_A_ and its proxy measures under conditions tested. Quantitative genetic parameters including high estimates of narrow‐sense heritability ($h_{n}^{2}>0.7;p<.05$) of WUE_A_ and related traits emphasized the genetic potential of the key trait combination for selecting PRG for improved drought tolerance. Research findings highlight the relative importance of WUE_A_ and its proxy measures in the broad definition of PRG drought tolerance for breeding purposes.

## Graphical abstract

Our results highlight the importance of selecting perennial ryegrass for agronomic water‐use efficiency (WUEa) at the whole‐plant level over its proxy measures for forage breeding purposes. The result was generated by key drought‐tolerant trait associations linked to WUEa and reinforced by its higher genetic potential and repeatability compared with those of surrogate measures under the conditions tested.

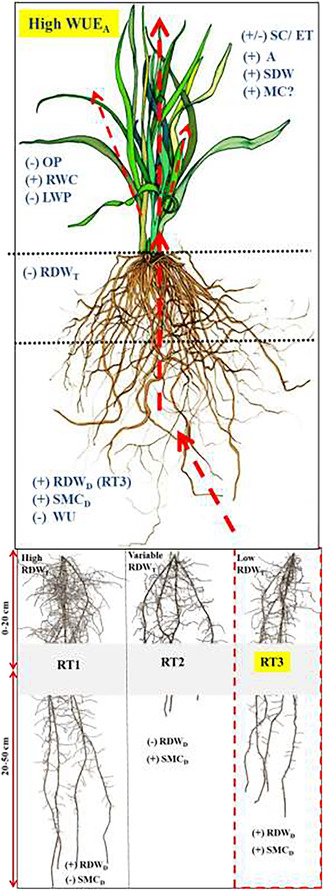

## 1 INTRODUCTION

*Lolium perenne* L. (perennial ryegrass; PRG) is the most widely grown pasture grass species in the world's temperate regions (Cyriac et al., 2018; McDonagh et al., 2016; Sampoux et al., 2011). However, soil drought in summer restricts its growth causing seasonal fluctuations in pasture productivity (Avramova et al., 2019; Cyriac et al., 2018; Lee et al., 2012). Hence, germplasm screening for drought tolerance traits has long been prioritized in PRG breeding programs (Lee et al., 2012; McDonagh et al., 2016; Sampoux et al., 2011). Over 10 decades of breeding, pasture plants have been screened for yield and phenotypic characteristics through visual scoring (Hatier et al., 2014; Lee et al., 2012). Nevertheless, while important to farmers, yield is one of the least useful selection criteria for drought stress tolerance due to morpho‐physiological complexities behind the existing gap between potential and actual yields of plants under stress conditions (Blum, 2005; Blum & Tuberosa, 2018). This association between drought tolerance and yield penalty has resulted in productivity traits generally being given priority within the plant breeding framework (Blum, 2011). On account of frequent and prolonged dry periods linked to climate change, water‐use efficiency (WUE) of PRG in summer has become important for maintaining year‐round productivity. Given that WUE is an important index in drought research but historically a less‐researched topic in forage breeding programs, this study was designed to address this research gap.

Drought causes an imbalance between the evapotranspiration demand of a plant and the actual soil water uptake, thus generating a hydraulic signal that leads to both synergistic and antagonistic changes in plants' physiology and metabolism (Avramova et al., 2019; Ferguson et al., 2018). Characteristics such as productivity and survival under drought are governed by a specific combination of dehydration avoidance or tolerance traits that optimize the water economy of crop plants (Avramova et al., 2019; Blum, 2005, 2009), including PRG (Weerarathne et al., 2021). Agronomic or whole‐plant level WUE (WUE_A_) is expressed as a plant's total dry matter production per unit of water used in a defined time period (Avramova et al., 2019; Blankenagel et al., 2018; Blum, 2005, 2009). However, instantaneous WUE, the ratio between the net assimilation and evapotranspiration (WUE_AET_) or stomatal conductance (WUE_ASC_), is often considered as a direct measure of the basic physiological response to drought (Avramova et al., 2019; Condon, 2020; Mininni et al., 2022; Seibt et al., 2008). Furthermore, WUE extrapolated from carbon isotope discrimination (Δ^13^C) or WUE derived from δ^13^C data (intrinsic WUE; WUE_i_) are used to characterize long‐term trends in the internal regulation of carbon and water uptake and water loss of drought‐stressed plants (Avramova et al., 2019; Mininni et al., 2022; Seibt et al., 2008). Hence, careful selection of drought‐responsive morpho‐physiological trait combinations is important when WUE, in any form, is considered as the key to drought tolerance (Blum, 2005, 2009; Weerarathne et al., 2021). Meanwhile, WUE_AET_, WUE_ASC_, and WUE_i_ and some other morpho‐agronomic and physiological measures have been used as easily measurable proxy data instead of the time‐consuming and laborious‐to‐measure WUE_A_ in many studies (Feldman et al., 2018; Moghaddam et al., 2013), including those on forage and pasture species (Ebdon & Kopp, 2004; Ghannoum et al., 2002; Read et al., 1991). When photosynthesis (A) is constant, reduced stomatal conductance (SC) leads to reduced Δ^13^C and subsequently, high WUE_i_ that may generate a link between WUE_i_ and WUE_ASC_ or WUE_AET_ (Avramova et al., 2019; Seibt et al., 2008) and differences may be expressed at the whole‐plant level WUE (WUE_A_). This is the logical basis upon which WUE_i_, WUE_ASC_, or WUE_AET_ are proposed as proxy measures of WUE_A_ (Seibt et al., 2008), although some studies have noted that the exact relationship between WUE_i_ and WUE_A_ is not yet fully defined (Blum, 2005, 2011; Condon, 2020). Given this tendency to use proxy data for WUE_A_, it is important to understand to what extent such proxy measures (with or without supportive trait data) truly capture the whole‐plant level WUE signal in a PRG plant under soil drought conditions, specifically for pasture selection and breeding purposes.

Information on the magnitude of genetic variation and the ability to predict genetic gain for selection traits will enhance the efficacy of plant breeding strategies (Acquaah, 2015). Moreover, the availability of quantitative genetic estimates derived in a specific stress environment targeted by a breeding program is an important step toward identifying appropriate selection traits to achieve genetic gain (Acquaah, 2015; Rutkoski, 2019). However, only a few studies have focused on estimating quantitative genetic parameters for WUE and related traits in PRG under imposed drought (Johnson et al., 1990; Johnson & Asay, 1993; Thomas, 1990). This may be partly due to the fact that Blum (2005, 2009) argued that selection for WUE, in any form, will likely result in unfavorable correlated trait responses, such as reduced plant size (i.e., when less water use results in high WUE) or increased water use (i.e., when high yield drives high WUE), and thus held that WUE is not a suitable selection trait for improving drought tolerance in annual field crops through breeding. As a result of these concerns, Blum (2009) made a distinction between WUE and an alternative strategy, “efficient use of water” (EUW) where the key principle of EUW is that crops shall receive a sufficient amount of water to keep up with an active growth and maximum yield even in a water deficit condition. In contrary to EUW, some plant species that exhibit high WUE may maintain growth and yield primarily through adjusted morpho‐physiology at reduced water supply. Given that, research evidence to clarify the roles of WUE_A_ including its proxy measures and related morpho‐physiological traits in improving the drought tolerance of PRG by breeding is currently lacking.

To address these knowledge gaps, the experiment reported here (i) assessed inter‐relationships among WUE, other plant water relations traits, and morpho‐physiological trait responses aligned with improved PRG drought tolerance; (ii) measured WUE_A_, WUE_i,_ WUE_AET_, and WUE_ASC_ simultaneously on the same PRG plants to determine their equivalence for describing plant drought tolerance based on WUE; and (iii) estimated quantitative genetic parameters of different WUE measures and related traits to understand their usefulness in improving PRG drought tolerance by breeding. These aims are designed to improve our understanding of the contribution of plant water relations traits to drought tolerance and to provide plant breeders with relevant information to inform decisions on selection criteria in plant breeding programs targeting improved drought tolerance.

## 2 MATERIALS AND METHODS

### 2.1 Overview and plant materials

The main experiment included 36 half‐sib families, carrying the AR37 endophyte, sampled from an advanced generation commercial breeding pool. There were five randomly sampled genotypes from each half‐sib family, two clonal replicates of each genotype, and a further 40 plants (20 clonal replicates each of two check plants A and B) included as checks to adjust for spatial variation (see Figure S1), making a total of 400 plants in the experiment. The half‐sib families were generated from a polycross isolation of 113 plants, randomly sampled from the advanced PRG breeding population designated Pop II (Faville et al., 2018; Gagic et al., 2018). Pop II was originally generated from a cross between a mid‐season‐flowering New Zealand cultivar and Spanish ecotypes that underwent recurrent selection for vigor, disease tolerance, and endophyte transmission (Gagic et al., 2018).

### 2.2 Experimental setup and drought treatment

The experiment was conducted in a glasshouse at the Plant Growth Unit, Massey University, New Zealand (40.3709° S, 175.6303° E, 35 m a.m.s.l.; Weerarathne, 2021). Plants were grown in pots made from sections of PVC pipe (10 cm diameter, 55 cm length), lined with polythene plastic perforated at the bottom to allow drainage and aeration. Saucers under each pot allowed for reabsorption rather than escape by drainage after watering. This planting system allowed root development similar to that in field swards. The soil used was from the A horizon of an “Egmont Black Loam” (a Typic Distrandept under USDA nomenclature; Perrott & Sarathchandra, 1987; Weerarathne et al., 2018, 2021). Pots were packed to a standard weight of 3.7–3.9 kg air‐dried soil per pot. When soil samples were tested on a pressure plate apparatus at −0.01, −0.1, and −1.5 MPa negative pressure, the gravimetric soil moisture content (SMC) was 66%, 46%, and 36%, respectively, indicating a capacity to supply >1000 mL water to each plant during an experiment dry‐down cycle from field capacity (FC) to −1.5 MPa.

PRG seedlings of half‐sib family groups were grown at FC for 8 weeks to a size of ≥10 tillers in a nursery and then divided into two clonal replicates of five tillers (including one primary and 3–4 secondary and tertiary tillers) for transplanting into pots. Pots were placed in a row‐column design with border plants in the glasshouse and time allowed for root system development, as indicated by leading roots reaching the bottom of pots before the drought treatment started (see Figure S1).

After an 84‐day establishment period near FC, all plants were defoliated to a similar height and maintained at 85%–95% FC (MS1) until the next defoliation 35 days later. Then, the watering interval was progressively increased, and the water volume decreased until the soil reached 55%–65% of FC (MS2) as determined by weighing the pots individually on an electronic balance to 1 g precision. Water was manually added 2–4 times weekly as required to keep all the pots at a constant weight and water use of each potted plant was recorded (Note: Soil samples used for filling the pots, when tested on a pressure plate apparatus at −0.01, −0.1, and − 1.5 MPa, had gravimetric soil moisture of 66%, 46%, and 36%, respectively). After 28 days at MS2, plants were defoliated again and maintained at 45%–55% FC (MS3) for another 14 days. Thus, three sets of detailed measurements of plant morpho‐physiological traits were carried out on all plants within 4‐day time windows at each of M1, M2, and M3 phases, for all the plants as described in Tables 1 and 2 below. After the last defoliation, irrigation was completely withdrawn, and postdefoliation regrowth was scored (see Table 1) after 7 days.

**TABLE 1:** Drought‐responsive water relations traits measured in the perennial ryegrass half‐sib family population (*n* = 400) throughout the current experiment (“†” denotes the measurements taken in a subset (*n* = 200) of the original population).

	Trait	Measurement methods	Measurement stage		
			1	2	3
1	RWC	Calculated using the method devised by (Barrs & Weatherly, 1962).	√	√	√
2	OP	Measured by the dew‐point method (Turner, 1981) using Wescor C–52 sample chambers and HR–33 T microvolt meter (Wescor). Samples of youngest fully expanded leaf snap‐frozen in liquid N_2_ and stored at −80°C until analysis.	√	√	√
3	LWP	Measured at predawn (between 3 a.m. and 6 a.m.) in the youngest fully expanded leaf of representative tillers using a Scholander's Pressure Chamber (Soil Moisture Equipment Corp.) (Turner, 1981).	√	√	√
4	SMC_T_	Determined by calculating the percentage of soil moisture (weight basis) at the 10–20 cm depth of the soil column of each pot: Oven‐dry weight of each soil sample was taken (at 105°C for 24 h) to a constant fresh weight	No	No	√
5	SMC_D_	Determined by calculating the percentage of soil moisture (weight basis) at the 35–45 cm depth of the soil column of each pot: Oven‐dry weight of each soil sample was taken (at 105°C for 24 hours) to a constant fresh weight	No	No	√
6	FASW	Nondestructively determined by calculating the percentage fraction of readily available soil moisture on the pot weight basis; 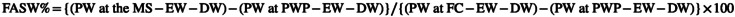 where, DW, dry soil weight; EW, empty pot weight; FC, field capacity; MS, measurement stage; PW, pot weight; PWP, permanent wilting point.	√	√	No
7	WUE_A_	Calculated by using integrated data of plant‐soil moisture uptake and evapotranspiration from pot weights and irrigated water and shoot dry matter recorded at each MS.	√	√	√
8 9	WUE_ASC_ WUE_AET_	Recorded Pn, SC, and ET data were used in the calculations of instantaneous WUE (Seibt et al., 2008).	√	√	√
9	WUEi^†^	Derived from Δ^13^Cdata using equations 1, 2 and 5 of (Barbosa et al., 2010) and leaf samples from M1 and M2a; indicates leaf‐level relationships, cumulative for an undefined preceding time.	√	√	No

Abbreviations: FASW (%, w/w), remaining fraction of soil available water; LWP (MPa), predawn leaf water potential; OP (MPa), leaf osmotic potential; RWC (%), leaf relative water content; SMC_D_ (%, w/w), gravimetric soil moisture content at deeper soil layers (40–50 cm); SMC_T_ (%, w/w), gravimetric soil moisture content at top soil layers (10–20 cm); WUE_A_ (g H_2_O g^−1^ DW), agronomic water‐use efficiency; WUE_ASC_ and WUE_AET_, instantaneous WUE (gas exchange‐based [μmol mol^−1^]); WUEi^†^, intrinsic WUE (Δ^13^C‐based [μmol mol^−1^]).

**TABLE 2:** Drought‐responsive morpho‐physiological traits measured in the perennial ryegrass half‐sib family population (*n* = 400) throughout the current experiment (‘†’ denotes the measurements taken in a subset (*n* = 200) of the original population).

	Trait	Measurement methods	Measurement stage		
			1	2	3
1	SDW	Shoot samples clipped 5 cm above soil surface consecutively and dried 48 h in an air‐draft oven at 80°C.	√	√	√
2	C:N^†^	Generated by elemental analysis in conjunction with Δ^13^C determination (Wittmer et al., 2008).	√	√	No
3	RDW_T_	Root mass recovered from 0 to 20 cm depth, 7 days after M2b, oven‐dried 48 h at 80°C.	No	No	√
4	RDW_D_	Root mass recovered from 20 to 50 cm depth, 7 days after M2b, oven‐dried 48 h at 80°C.	No	No	√
5	RGS	Scored after 7 days of recovery regrowth following M2b; based on the live tiller number as a percentage of total tillers; 0 = 0–5%, 1 = 6–25%, 2 = 26–45%, 3 = 46–65%, 4 = 66–85%, 5 = 86–100%.	No	No	√
6	TN	The total number of tillers of each plant was manually counted at each defoliation.	√	√	√
7 8 9	A SC ET	(7) Photosynthesis, (8) stomatal conductance, and (9) evapotranspiration of fully expanded youngest leaves were measured using a portable photosynthesis meter (LiCor6400, LiCor Inc., USA) under artificial, saturating photon flux density (1000 molm^2^ s^−1^) at an ambient CO_2_ concentration of 400 μmolCO_2_mol^−1^ (leaf chamber temperature and relative humidity: 26°C and 60%, respectively). Note: Measurements were performed for two replicates of each genotype from 10.00 am to 12.00 noon and 2.00 to 4.00 pm for four consecutive sunny days at the end of each MS and data were recalculated according to actual leaf area fitted in to the chamber and standardized by employing “subtract mean and divide by standard deviation” option in MINITAB14 to eliminate day and time effect.	√	√	√
11	Δ^13^C^†^	Carbon isotopic data from 0.7 to 0.8 mg of ground material of each sample were presented as δ^13^C (‰): δ^13^C (‰) = (R plant sample/R standard) –1 × 1000 where R is the ^13^C/^12^C ratio, with the standard; Vienna Pee Dee Belemnite standard (Wittmer et al., 2008; Wittmer et al., 2010). Each sample was measured against a laboratory working standard CO_2_ gas, which was previously calibrated against an IAEA secondary standard (IAEA–CH6, accuracy of calibration 0.06% SD) (Wittmer et al., 2010). Carbon isotope discrimination (∆^13^C) of a plant to atmospheric air was calculated as: ∆^13^C (‰) = (δa – δp)/ (1+ δp) where δa is the δ^13^C of the atmospheric air; approximately −8‰ and δp is the δ^13^C of the plant sample (Barbosa et al., 2010). Wheat flour (C/N 21:6) as solid internal laboratory standard (SILS) was calibrated against these references. One SILS was measured after every tenth sample and the precision for sample repeats was considered to be smaller than 0.2‰ for δ^13^C (Wittmer et al., 2008).	√	√	No

Abbreviations: A (μmol m^−2^ s^−1^), photosynthesis; C:N^†^, carbon: nitrogen ratio; ET (mmol m^−2^ s^−1^), evapotranspiration; RDW_D_ (g plant^−1^), deep root dry weight (20–50 cm); RDW_T_ (g plant^−1^), top root dry weight (0–20 cm); RGS, postdefoliation regrowth score; SC (mmol m^−2^ s^−1^), stomatal conductance; SDW (g plant^−1^), shoot dry weight; TN, tiller number; Δ^13^C^†^, carbon isotope discrimination of oven‐dried (1 h at 105°C and 48 h at 60°C) fully expanded youngest leaf samples from representative tillers.

### 2.3 Trait measurements

**FIGURE 1:**
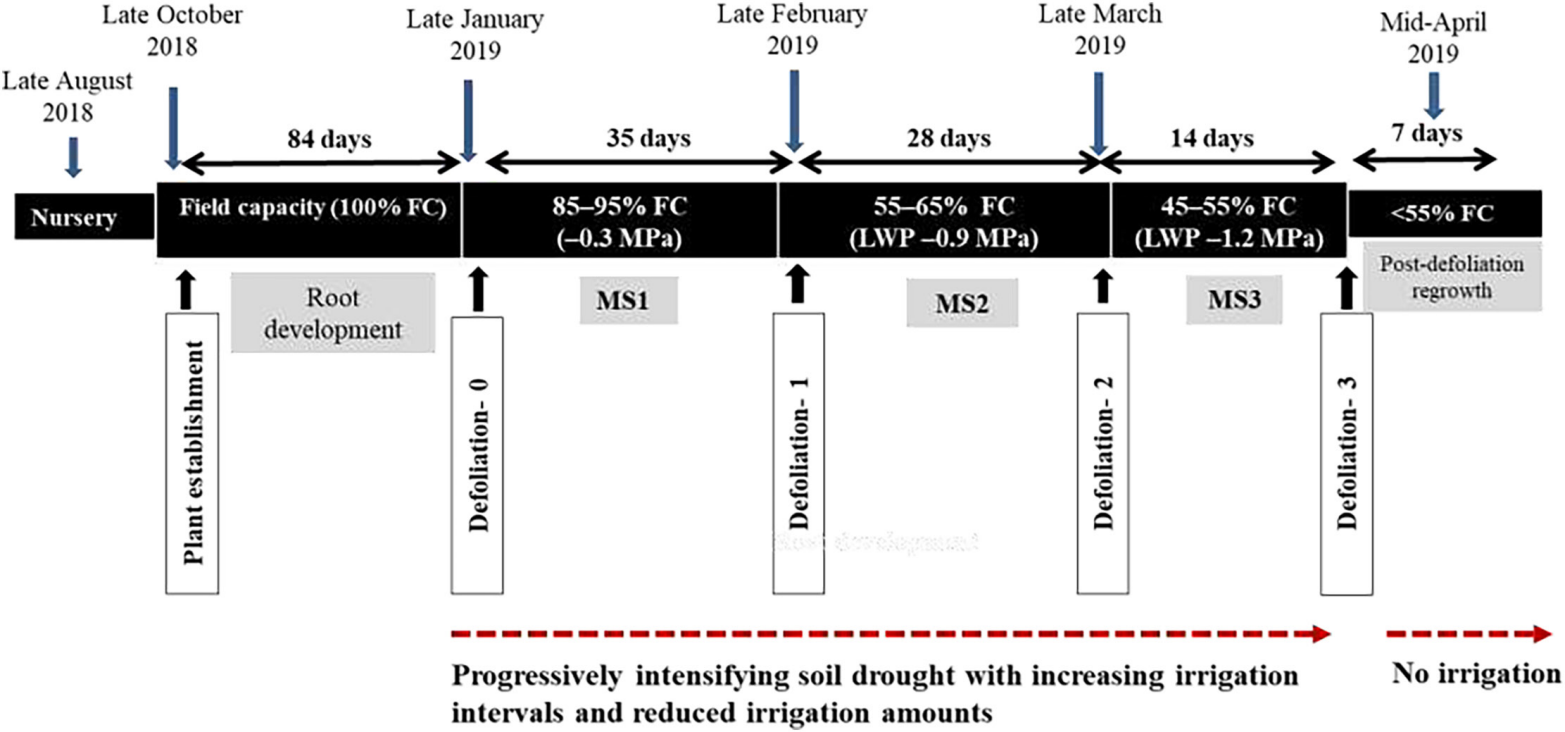
Treatment structure of the current experiment. MS1, MS2, and MS3, Measurement stages 1, 2, and 3 or mild drought, moderate drought, and intense drought, respectively; LWP, Predawn leaf water potential.

Leaf water relations traits measured on the experimental plants include leaf relative water content (RWC), leaf osmotic potential (OP), predawn leaf water potential (LWP), gravimetric soil moisture content at 10–20 and 40–50 cm depths (SMC_T_ and SMC_D_, respectively), remaining fraction of soil available water (FASW), agronomic water‐use efficiency (WUE_A_), photosynthetic gas exchange‐based instantaneous WUE based on stomatal conductance or evapotranspiration (WUE_ASC_ and WUE_AET_), and (δ^13^C‐based intrinsic WUE [WUEi; see Table 1]). The set of morpho‐physiological traits measured included shoot dry weight (SDW), carbon: nitrogen ratio (C:N), surface root dry weight at 0–20 cm soil depth (RDW_T_), deep root dry weight at 20–50 cm soil depth (RDW_D_), postdefoliation regrowth score (RGS), tiller number (TN), photosynthesis (A), stomatal conductance (SC), evapotranspiration (ET), and carbon isotope discrimination (Δ^13^C; see Table 2). SMC_T,_ SMC_D_, RDW_T_, RDW_D_, and RGS were evaluated only once using destructive measurements at the end of the experiment, and all other traits (excluding carbon isotope data; see below) were recorded (or samples stored for later measurement) within a 4‐day time window at least twice on the full set of plants (36 half‐sib families and 40 check plants; *n* = 400) at the end of each successive postdefoliation regrowth phase or measurement stages 1, 2, and 3 (MS1, MS2, and MS3, respectively) of a progressively intensifying water deficit (Figure 1).

A subset of samples taken from the 400 plants in the main experiment was used to evaluate δ^13^C (‰) of leaf tissue by isotope ratio mass spectrometry (IRMS) of leaf tissue collected at MS1 and MS2 to compare and contrast four measures of WUE: WUE_A_, WUE_AET_, WUE_ASC_, and WUE_i_. Genotypes for IRMS analysis of stored leaf tissue were selected on a “stratified random” basis by ranking the 36 half‐sib family means of the main experiment in ascending order of WUE_A_ and selecting every alternate value excluding two near‐equivalents. The sample subset therefore comprised two clonal replicates of five genotypes of 16 families plus the 40 check plants (*n* = 200), but largely retained the statistical variance properties of the larger dataset with respect to WUE_A_. Stored leaf samples for IRMS were first re‐dried at 60°C for 48 h in an air‐draft oven, powdered, and then submitted for carbon isotope analysis to derive carbon isotope discrimination (Δ^13^C), δ^13^C (‰), and carbon: nitrogen ratio (CN) data. For these determinations, the samples collected at Massey University, New Zealand, were analyzed at Technical University of Munich, Germany, using an elemental analyzer (NA 1110; Carlo Erba) interfaced (ConFlo III; Finnigan MAT) to an isotope ratio mass spectrometer (Delta Plus; Finnigan MAT) (Tables 1 and 2).

### 2.4 Statistical analyses

An ANOVA model within SAS Proc GLM was used to examine genotypic and half‐sib family means of traits measured at MS1, MS2, and MS3 (Table 3). Trait means were generated using Duncan's new multiple range test (Assumption: Data were normally distributed around the sample mean). Pearson correlation analyses were performed on measured water relations and morpho‐physiological data as an initial exploration of trait associations within and among MS1, MS2, and MS3 (Table 4; see Tables S1a and S1b). Two major principal component analyses following methodology of Jolliffe (2003) were compiled for original data: PCA1 included data from MS3 for 180 genotypes (all measured trait means as averaged for the two clonal replicates of 360 test plants excluding 40 check plants of the full set of plants) (Table 5). PCA2 included data for 80 genotypes (all measured trait means including WUE proxies at MS2 as averaged for two clonal replicates [160 test plants] of the subset selected for Δ^13^C determination, excluding 40 check plants) to establish all‐inclusive key drought‐response patterns and to discern the relative importance and interrelationship of different WUE measures in PRG drought tolerance at MS2 (Table 6).

**TABLE 3:** Trait means ± standard error of means (SE) and statistical probabilities (*p*) of half‐sib families (HSF) and within‐HSF (Gen) effects of the water‐use efficiency measures and related morpho‐physiological traits tested HSF population at two to three consecutive measurement phases in the imposed drought cycle; MS1 (field capacity; FC), MS2 (55%–65% FC; early drought), and MS3 (45%–65% FC; late drought), respectively.

	Min			Max			Mean ± SE & *p* values								
	MS1	MS2	MS3	MS1	MS2	MS3	MS1	*p* (HSF)	*p* (gen)	MS2	*p* (HSF)	*p* (gen)	MS3	*p* (HSF)	*p* (gen)
SDW	0.80	2.01	1.42	5.78	6.08	6.18	3.55 ± 0.04	***	*ns*	3.99 ± 0.04	***	***	4.16 ± 0.04	***	***
WUE_A_	301.38	133.75	135.05	978.18	993.09	998.84	528 ± 7.52	**	*ns*	505 ± 6.07	***	**	466 ± 5.93	***	***
TN	4.00	21.00	33.00	73.00	147.00	178.00	32 ± 0.63	***	*ns*	83 ± 1.24	***	***	89 ± 1.18	***	***
LWP	−0.77	−1.09	−1.44	−0.23	−0.60	−0.90	−0.4 ± 0.05	***	*ns*	−0.7 ± 0.03	***	***	−1.1 ± 0.06	***	***
RWC	74.09	35.30	42.27	99.59	68.62	76.58	86 ± 0.25	**	*ns*	58 ± 0.26	***	*ns*	57 ± 0.37	***	***
OP	−1.95	−3.45	−3.70	−0.79	−1.34	−1.61	−1.2 ± 0.09	**	*	−2.3 ± 0.26	***	**	−2.8 ± 0.29	***	***
FASW	60.06	41.95		89.95	67.93		72 ± 0.35	***	*ns*	55 ± 0.25	***	*ns*			
A	9.01	0.02	1.01	16.70	0.15	5.96	13. ± 0.08	***	*ns*	7 ± 0.06	***	*ns*	4. ± 0.03	***	*ns*
SC	0.16	4.04	0.02	0.35	10.65	0.07	0.23 ± 0.00	***	*ns*	0.07 ± 0.00	***	*	0.04 ± 0.00	***	*
ET	0.15	0.39	0.17	2.94	1.96	1.27	0.99 ± 0.03	***	*ns*	0.99 ± 0.02	*	*ns*	0.74 ± 0.01	***	*ns*
WUE_ASC_	43.13	54.57	58.57	68.22	243.59	229.09	57 ± 0.25	***	*ns*	115 ± 1.20	**	*ns*	117 ± 1.03	*ns*	*ns*
WUE_AET_	3.78	3.34	2.91	67.89	17.86	24.67	18 ± 0.62	***	*ns*	8 ± 0.14	**	*ns*	6 ± 0.15	*	*ns*
WUE_i_ ^†^	24.57	34.13		85.27	97.54		56 ± 0.94	***	*ns*	68 ± 0.87	***	*ns*			
RDW_T_		0.47	0.47		3.17	3.17							1.83 ± 0.02	***	*ns*
RDW_D_		0.01	0.01		2.14	2.14							0.88 ± 0.03	***	***
SMC_T_			14.05			43.21							25 ± 0.17	***	***
SMC_D_			19.02			42.98							27 ± 0.23	***	***
RGS			0			5							3 ± 0.05	***	**

Abbreviations: A (μmol m^−2^ s^−1^), photosynthesis; ET (mmol m^−2^ s^−1^), evapotranspiration; FASW (%, w/w), remaining fraction of soil available water; LWP (bars), predawn leaf water potential; OP (bars), leaf osmotic potential; RDW_D_ (g plant^−1^), deep root dry weight (20–50 cm); RDW_T_ (g plant^−1^), top root dry weight (0–20 cm); RGS, postdefoliation regrowth score.; RWC (%), leaf relative water content; SC (mmol m^−2^ s^−1^), stomatal conductance; SDW (g plant^−1^), shoot dry weight; SMC_D_ (%, w/w), gravimetric soil moisture content at deeper soil layers (40–50 cm); SMC_T_ (%, w/w), gravimetric soil moisture content at top soil layers (10–20 cm); TN, tiller number; WUE_A_ (g H_2_O g^−1^ DW), agronomic water‐use efficiency; WUE_ASC_ and WUE_AET_, instantaneous WUE (gas exchange‐based [μmol mol^−1^]); WUEi^†^, intrinsic WUE (Δ^13^C‐based [μmol mol^−1^]).

*p < .1; **p < .05; ***p < .001; *ns*, *p* > .1. ^†^WUE_i_ was measured only in a subset (*n* = 200) of the original population.

**TABLE 4:** Pearson correlation coefficients between different measures of perennial ryegrass water‐use efficiency (i.e., WUE_A_, WUE_i_, WUE_AET_, and WUE_ASC_) and that against key plant‐soil‐water relations and morpho‐physiological traits of a subset of the original half‐sib family population (*n* = 200) as measured at the end of early drought stage (MS2; 55%–65% FC).

	WUE_i_	WUE_AET_	WUE_ASC_	WUE_A_	FASW	OP	RWC	LWP	SDW	TN	SC	ET	A	RDW_D_
WUE_i_		0.100*	0.120*	0.470**	0.421**	—	−0.172*	—	−0.561**	−0.400**	**−**0.222*	—	−0.330**	—
WUE_AET_			0.540**	0.190*	0.270**	−0.740**	−0.190*	—	−0.340**	−0.290**	−0.740**	−0.920**	−0.430**	—
WUE_ASC_				0.240*	0.211*	—	−0.231*	—	−0.244*	−0.221*	−0.730**	−0.420**	−0.221*	—
WUE_A_					0.190*	0.241*	−0.141*	0.210*	−0.601**	−0.511**	−0.151*	−0.101*	−0.162*	0.237*

Abbreviations: A (μmol m^−2^ s^−1^), photosynthesis; ET (mmol m^−2^ s^−1^), evapotranspiration; FASW (%, w/w), remaining fraction of soil available water; LWP (MPa), predawn leaf water potential; OP (MPa), leaf osmotic potential; RDW_D_ (g plant^−1^), deep root dry weight (20–50 cm); RWC (%); leaf relative water content; SC (mmol m^−2^ s^−1^), stomatal conductance; SDW (g plant^−1^), shoot dry weight; TN, tiller number; WUE_A_ (g H_2_O g^−1^ DW), agronomic water‐use efficiency; WUEA_ET_ and WUE_ASC_, instantaneous WUE (gas exchange‐based [μmol mol^−1^]); WUEi, intrinsic WUE (Δ^13^C‐based [μmol mol^−1^]).

* *p* < .05.

** *p* < .001.

**TABLE 5:** Principal component (PC) coefficients for the first three PCs generated by PCA1 of 14 morpho‐physiological and water relations traits, respectively, as obtained from 180 genotype means of two clonal replicates of 360 test plants (excluding check plants) of the half‐sib family population, measured at the end of the late drought stage (MS3; 45%–55% FC). As this was a destructive harvest, soil moisture (SMC) data were available for inclusion.

Eigenvalue	7.88	1.90	1.40
% variation explained	56	14	10
Cumulative % variance	56	70	80

	PC1	PC2	PC3
SDW	0.287	0.158	0.326
WUE_A_	−0.237	−0.087	−0.424
TN	0.188	−0.058	0.334
LWP	−0.299	−0.107	−0.224
RWC	0.326	0.158	−0.147
OP	−0.333	−0.122	0.137
A	0.315	−0.154	−0.087
SC	0.224	−0.457	−0.265
ET	0.229	−0.423	−0.283
RDW_T_	−0.114	0.562	−0.205
RDW_D_	0.301	0.304	−0.202
RGS	0.318	0.043	0.177
SMC_T_	−0.142	−0.298	0.480
SMC_D_	0.304	−0.049	−0.096

Abbreviations: A (μmol m^−2^ s^−1^), photosynthesis; ET (mmol m^−2^ s^−1^), evapotranspiration; LWP (MPa), predawn leaf water potential; OP (MPa), leaf osmotic potential; RDW_D_ (g plant^−1^), deep root dry weight (20–50 cm); RDW_T_ (g plant^−1^), top root dry weight (0–20 cm); RGS, postdefoliation regrowth score; RWC (%), leaf relative water content; SC (mmol m^−2^ s^−1^), stomatal conductance; SDW (g plant^−1^), shoot dry weight; SMC_D_ (%, w/w), gravimetric soil moisture content at deeper soil layers (40–50 cm); SMC_T_ (%, w/w), gravimetric soil moisture content at top soil layers (10–20 cm); TN, tiller number; WUE_A_ (g H_2_O g^−1^ DW), agronomic water‐use efficiency.

**TABLE 6:** Principal component (PC) coefficients for the first five PCs generated by PCA2 of 17 morpho‐physiological and water relations data, respectively, as obtained from 80 genotype means of two clonal replicates of 160 test plants (excluding check plants) of a subset of the original half‐sib family population, measured at the end of the early drought stage (MS2; 55%–65% FC).

Eigenvalue	8.10	1.10	1.82	1.15	1.03
% variation explained	48	12	11	7	6
Cumulative % variance	48	59	70	77	83

	PC1	PC2	PC3	PC4	PC5
WUE_i_	0.259	0.127	0.393	0.158	0.118
C:N	0.128	−0.245	0.314	0.465	−0.168
SDW	−0.332	−0.044	−0.122	0.147	−0.039
WUE_A_	0.283	0.195	0.126	−0.101	0.043
TN	−0.313	−0.024	−0.077	0.201	−0.033
LWP	0.063	−0.227	−0.029	−0.644	0.355
RWC	−0.250	0.100	−0.030	0.100	0.132
OP	0.054	−0.632	0.091	−0.032	−0.075
FASW	0.313	0.036	0.108	−0.156	−0.053
SC	−0.311	0.042	0.285	−0.113	−0.020
A	−0.309	−0.034	0.047	0.079	−0.079
ET	−0.264	0.088	0.393	−0.105	0.008
WUE_ASC_	0.241	−0.053	−0.314	0.248	−0.040
WUE_AET_	0.262	−0.092	−0.409	0.152	0.011
RDW_T_	0.070	−0.095	0.076	−0.301	−0.852
RDW_D_	0.023	0.614	−0.158	−0.080	−0.231

Abbreviations: A (μmol m^−2^ s^−1^), photosynthesis; C:N, carbon: nitrogen ratio; ET (mmol m^−2^ s^−1^), evapotranspiration; FASW (%, w/w), remaining fraction of soil available water; OP (MPa), leaf osmotic potential; RDW_D_ (g plant^−1^), deep root dry weight (20–50 cm); RDW_T_ (g plant^−1^), top root dry weight (0–20 cm); RWC (%), leaf relative water content; SC (mmol m^−2^ s^−1^), stomatal conductance; SDW (g plant^−1^), shoot dry weight; TN, tiller number; LWP (MPa), predawn leaf water potential; WUE_A_ (g H_2_O g^−1^ DW), agronomic water‐use efficiency; WUE_ASC_ and WUE_AET_, gas exchange‐based instantaneous WUE (μmol mol^−1^); WUEi, Δ^13^C‐based intrinsic WUE (μmol mol^−1^).

### 2.5 Quantitative genetic analyses

Analysis of variance was carried out to estimate the magnitude and significance of additive genetic variation among the half‐sib families at MS2 (Table 7) and MS3 (Table 8) for the traits of interest. The residual maximum likelihood (REML) procedure using a complete random linear model (Equation S1) was used to generate best linear unbiased predictor (BLUP) values (White & Hodge, 2013) and genetic variance components. Narrow‐sense heritability on a family mean basis (Tables 7 and 8), predicted genetic gain, genetic correlation (*r*_g_), and correlated response to selection for the measured traits were estimated (Table 9). These quantitative genetic analyses were performed using DeltaGen (v. 0.03) (Jahufer & Luo, 2018).

**TABLE 7:** Half‐sib family (HSF) mean narrow‐sense heritability ($h_{n}^{2}$), statistically significant (*p* < .05) variance components including genotype‐within‐HSFs$\;(\sigma_{f/s}^{2}$), among‐HSFs ($\sigma_{f}^{2}$), and residual error ($\sigma_{\varepsilon }^{2}$) and coefficient of variation (CV%) of traits of interest as estimated in the subset of HSF population (i.e., 80 genotypes from 16 HSFs and 40 check plants; *n* = 200) in early drought (MS2; 55%–65% FC).

Trait	$h_{n}^{2}$	Variance components			BLUP mean	CV%
		$\sigma_{f/s}^{2}$	$\sigma_{f}^{2}$	$\sigma_{\varepsilon }^{2}$		
SDW	0.75 ± 0.09	0.007 ± 0.030	0.145 ± 0.066	0.490 ± 0.058	4.06	17
TN	0.58 ± 0.16	*ns*	81.20 ± 40.07	588.19 ± 72.25	86	28
WUE_A_	0.61 ± 0.16	*ns*	2110 ± 1053	13,463 ± 1601	308.39	18
WUE_i_	0.76 ± 0.10	*ns*	31.96 ± 15.94	106.67 ± 13.84	67.75	15
C:N	0.63 ± 0.21	*ns*	0.079 ± 0.063	0.275 ± 0.042	8.52	6
LWP	*ns*	*ns*	*ns*	0.367 ± 0.085	−7.01	*ns*
RWC	*ns*	*ns*	*ns*	24.223 ± 3.140	53.00	*ns*
OP	0.82 ± 0.08	7.668 ± 2.554	6.054 ± 3.003	13.294 ± 1.809	−22.75	16
SC	*ns*	*ns*	*ns*	*ns*	0.065	23
A	0.59 ± 0.16	*ns*	0.167 ± 0.079	0.878 ± 0.113	7.21	13
ET	*ns*	*ns*	*ns*	0.171 ± 0.022	1.00	*ns*
WUE_AET_	*ns*	*ns*	*ns*	7.558 ± 0.968	114.98	*ns*
WUE_ASC_	*ns*	*ns*	*ns*	465.72 ± 47.71	8.33	*ns*
FASW	0.67 ± 0.12	*ns*	4.859 ± 2.411	23.824 ± 2.864	56.80	9

*Note*: *‘ns*’ stands for statistical nonsignificance.

Abbreviations: A (μmol m^−2^ s^−1^), photosynthesis; C:N, carbon: nitrogen ratio; ET (mmol m^−2^ s^−1^), evapotranspiration; FASW (%, w/w), remaining fraction of soil available water; LWP (MPa), predawn leaf water potential; OP (MPa), leaf osmotic potential; RWC (%), leaf relative water content; SC (mmol m^−2^ s^−1^), stomatal conductance; SDW (g plant^−1^), shoot dry weight; TN, tiller number; WUE_A_ (g H_2_O g^−1^ DW), agronomic water‐use efficiency; WUE_ASC_ and WUE_AET_, instantaneous WUE (gas exchange‐based [μmol mol^−1^]); WUEi, intrinsic WUE (Δ^13^C‐based [μmol mol^−1^]).

**TABLE 8:** Half‐sib family (HSF) mean narrow‐sense heritability ($h_{n}^{2}$), statistically significant (*p* < .05) variance components including genotype‐within‐HSFs ($\sigma_{f/s}^{2}$), among‐HSFs ($\sigma_{f}^{2}$), and residual error ($\sigma_{\varepsilon }^{2}$), and coefficient of variation (CV%) of traits as estimated in the original HSF population (i.e., 180 genotypes from 36 HSFs and 40 check plants; *n* = 400) at late drought (MS3; 45%–55% FC).

Trait	$h_{n}^{2}$	Variance components			BLUP mean	CV%
		$\sigma_{f/s}^{2}$	$\sigma_{f}^{2}$	$\sigma_{\varepsilon }^{2}$		
SDW	0.94 ± 0.04	0.406 ± 0.081	0.258 ± 0.120	0.126 ± 0.018	4.01	9
TN	0.87 ± 0.06	242.97 ± 57.73	121.44 ± 64.07	178.22 ± 23.55	85.00	16
RDW_T_	*ns*	0.044 ± 0.0174	*ns*	0.114 ± 0.016	1.82	*ns*
RDW_D_	0.87 ± 0.07	0.175 ± 0.038	0.080 ± 0.037	0.092 ± 0.013	0.71	23
RGS	0.89 ± 0.07	0.536 ± 0.115	0.241 ± 0.119	0.263 ± 0.038	3.00	16
LWP	0.87 ± 0.06	0.537 ± 0.145	0.388 ± 0.187	0.594 ± 0.078	−11.00	7
RWC	0.91 ± 0.05	27.056 ± 5.435	*ns*	9.190 ± 1.210	56.08	5
OP	0.96 ± 0.02	17.913 ± 3.405	8.928 ± 4.028	3.638 ± 0.484	−26.17	7
A	0.84 ± 0.10	0.174 ± 0.038	*ns*	0.089 ± 0.012	3.93	8
SC	0.60 ± 0.27	–	–	–	0.04	17
ET	0.60 ± 0.30	0.036 ± 0.010	0.006 ± 0.007	0.039 ± 0.005	0.73	27
WUE_ASC_	*ns*	267.34 ± 61.49	*ns*	239.44 ± 31.98	106.26	*ns*
WUE_AET_	*ns*	5.621 ± 1.302	*ns*	5.025 ± 0.681	6.49	*ns*
WUE_A_	0.92 ± 0.04	7912 ± 1688	4410 ± 2165	3644 ± 497	472.08	13
SMC_T_	*ns*	5.888 ± 1.34	*ns*	5.083 ± 0.705	25.17	*ns*
SMC_D_	0.86 ± 0.09	6.132 ± 1.556	4.313 ± 2.080	5.887 ± 0.828	26.25	9

*Note*: *‘ns*’ stands for statistical nonsignificance.

Abbreviations: A (μmol m^−2^ s^−1^), photosynthesis; ET (mmol m^−2^ s^−1^), evapotranspiration; LWP (MPa), predawn leaf water potential; OP (MPa), leaf osmotic potential; RDW_D_ (g plant^−1^), deep root dry weight (20–50 cm); RDW_T_ (g plant^−1^), top root dry weight (0–20 cm); RGS, postdefoliation regrowth score; RWC (%), leaf relative water content; SC (mmol m^−2^ s^−1^); stomatal conductance; SDW (g plant^−1^), shoot dry weight; SMC_D_ (%, w/w), gravimetric soil moisture content at deeper soil layers (40–50 cm); SMC_T_ (%, w/w), gravimetric soil moisture content at top soil layers (10–20 cm); TN, tiller number; WUE_A_ (g H_2_O g^−1^ DW), agronomic water‐use efficiency; WUE_ASC_ and WUE_AET_, instantaneous WUE (gas exchange‐based [μmol mol^−1^]).

**TABLE 9:** Predicted genetic gain (%∆G_c_) of the morpho‐physiological and water relations traits as estimated in a subset of the tested half‐sib family population at the end of early drought (MS2; 55%–65% FC) and late drought (MS3; 45%–55% FC) through among‐family‐selection (AFS) and among‐and‐within‐family‐selection (AWFS) at 30% selection pressure.

	%∆G_c_ (AFS)		%∆G_c_ (AWFS)	
	MS2	MS3	MS2	MS3
SDW	2.34	**3.57**	4.69	7.14
TN	2.30	**3.44**	4.59	6.87
RGS	‐	**3.92**	‐	7.83
RDW_D_	‐	**9.08**	‐	18.18
FASW	0.92	‐	1.84	‐
SMC_D_	‐	2.14	‐	4.27
OP	2.81	**3.04**	5.62	6.08
RWC	‐	1.48	‐	2.97
LWP	‐	1.58	‐	3.16
WUE_A_	**3.38**	**3.89**	6.76	7.79
WUEi	2.10	‐	4.20	‐
C:N	0.75	‐	1.50	‐
SC	‐	2.29	‐	4.60
ET	‐	‐	‐	‐
A	0.99	1.49	1.84	2.98

*Note*: Noticeable ∆G_c_ estimates are presented in bold numbers.

**Abbreviations:** A (μmol m^−2^ s^−1^), photosynthesis; C:N, carbon: nitrogen ratio; ET (mmol m^−2^ s^−1^), evapotranspiration; FASW (%, w/w), remaining fraction of soil available water; LWP (MPa), predawn leaf water potential; OP (MPa), leaf osmotic potential; RDW_D_ (g plant^−1^), deeper root dry weight (20–50 cm); RGS, postdefoliation regrowth score; RWC (%), leaf relative water content; SC (mmol m^−2^ s^−1^), stomatal conductance; SDW (g plant^−1^), shoot dry weight; SMC_D_ (%, w/w), gravimetric soil moisture content at deeper soil layers (40–50 cm); TN, tiller number; WUE_A_ (g H_2_O g^−1^ DW), agronomic water‐use efficiency; WUE_i_, intrinsic WUE (Δ^13^C‐based [μmol mol^−1^]).

## 3 RESULTS

### 3.1 Means, phenotypic variation, and correlation coefficients of WUE measures and associated traits.

Among‐half‐sib family differences for most trait measurements were significant (*p* < .0001) at each consecutive measurement stage of the progressively intensifying drought cycle (Table 3). Even so, at MS1 compared with MS2 and MS3, within‐half‐sib family variation was not significant for most traits (*p* > .05) with an increasing trend in statistical significance of variation toward the end of the drought cycle (Table 3). An exception was that gas exchange measurements and gas exchange‐related WUE measures were not significant at MS3 (*p* > .05; Table 3). The strongest correlations observed at MS3 were in WUE_A_‐OP, OP‐RWC, OP‐RGS, OP‐ RDW_D_, OP‐A, and RWC‐RDW_D_ trait pairs (>0.7; *p* < .001; Table 4).

Average WUE_A_ values at MS1, MS2, and MS3 showed a declining trend (indicating increased efficiency of plant water use) toward the end of the experiment, along with a corresponding declining trend in average A, SC, ET, WUE_ASC,_ and WUE_AET_ and an increasing trend in SDW from MS1 to MS3 (Table 3) and a decrease in WUE_i_ (i.e., a higher numerical value) from MS1 to MS2. However, WUE_A_ and gas exchange measurements were weakly correlated at all three measurement stages (Table 4) while A, SC, and ET and SDW were highly correlated with each other at both MS2 and MS3 at *p* < .05 (see Table S1a and S1b). At MS2, WUE_i_ of the subset of plants for which Δ^13^C was determined was moderately correlated with WUE_A_, FASW, A, and SDW and weakly correlated with WUEA_ET_, WUE_ASC_, SC, and RWC at *p* < .05 (Table 4).

As expected, WUEA_ET_ and WUE_ASC_ were highly correlated with photosynthetic gas exchange measurements (Table 4).

### 3.2 Major drought‐response trait associations.

**FIGURE 2:**
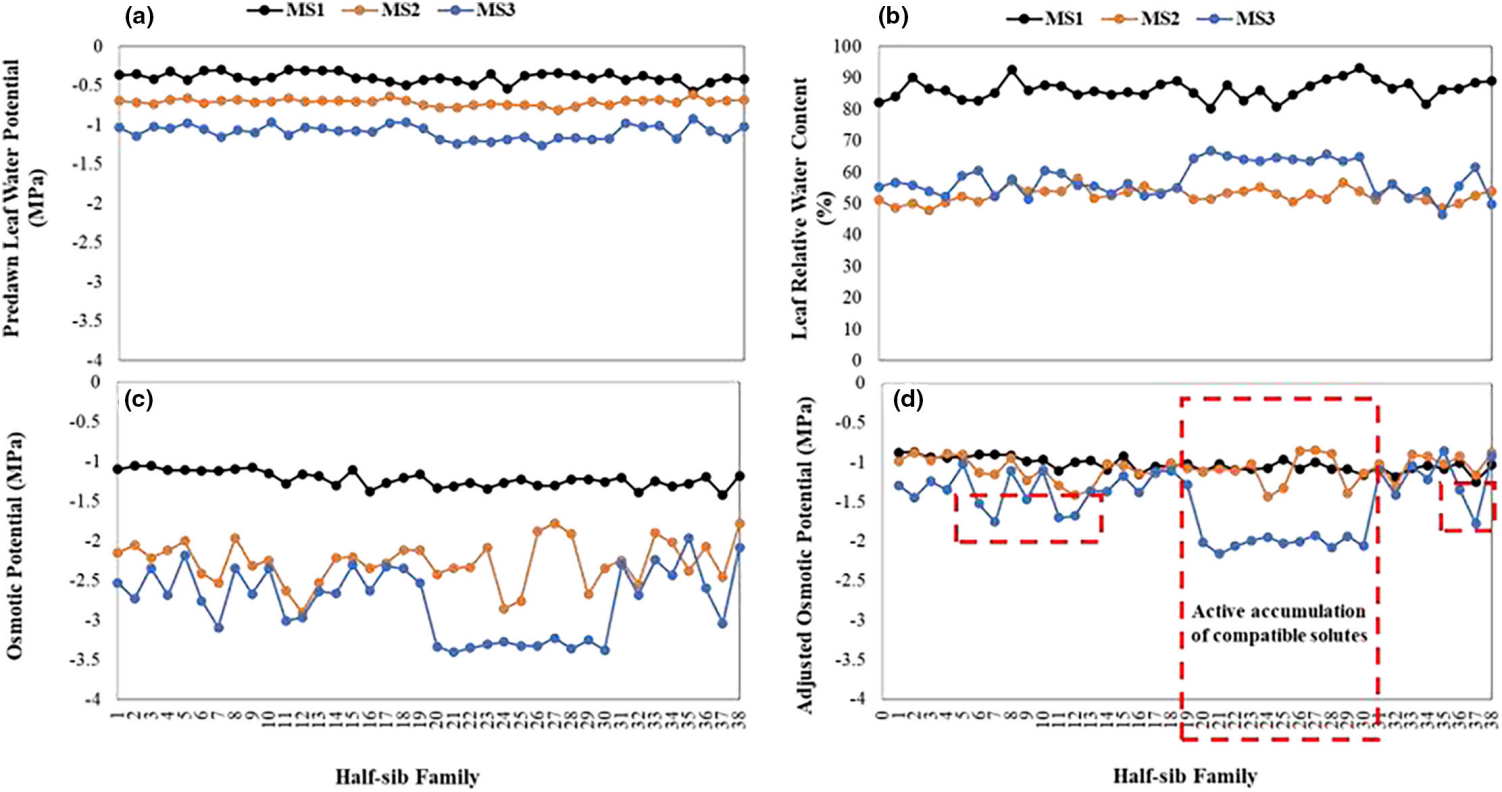
(a) Predawn leaf water potential, (b) leaf relative water content (RWC), (c) leaf osmotic potential (OP), and (d) adjusted OP in 400 perennial ryegrass half‐sib family plants tested at the measurement stages 1, 2, and 3 (MS1, MS2, and MS3, respectively) in the current experiment. To understand the active accumulation of compatible solutes, adjusted OP was also calculated. For this, the adjusted OP or solute potential (*Ψ*_*S*_100) was estimated using the equation; $\varphi_{s}100=\varphi_{s}\frac{\mathrm{RWC}-0.1}{1-0.1}$, where *φ*_*s*_ is OP and 0.1 is the estimated water content in apoplast tissue as described in Cyriac et al. (2018).

To visualize the major data variation for the traits measured in the full set of PRG half‐sib families at MS3, the first three PCs explaining 79.8% of data variation (eigenvalue >1) of PCA1 were selected (Table 5). PC1 represented the genotype scores for the primary drought‐response trait association explaining 56% of data variation, as identified among the 180 genotypes from 36 half‐sib families. In PC1, high plant yield (positive coefficients for SDW, RGS, TN) was linked to enhanced WUE_A_ (PC coefficient −0.237), RWC (PC coefficient 0.326), more negative OP (PC coefficient −0.333), enhanced gas exchange (positive PC coefficients for A, SC and ET), deep rootedness (PC coefficients for RDW_T_ and RDW_D_ were −0.114 and 0.301, respectively), and conservation of soil moisture at deep layers or high SMC_D_ (PC coefficient 0.304) (Table 6). PC2 identified variation among individual genotypes based on high root biomass (PC coefficients for RDW_T_ and RDW_D_ were +0.562, and +0.304, respectively) linked to decreased SMC_T_ with modestly positive coefficients for SDW explaining 14% of data variation. Paradoxically, this PC had negative coefficients for gas exchange and net assimilation (Table 5). Figure 2 depicts the major plant water relations that were governed by more negative OP (including adjusted OP; Cyriac et al., 2018) and were strongly linked to the ‐rooting feature of both the trait associations identified by PC1 and PC2. PC3, explaining 10% of data variation, explicated a drought‐response mechanism involving low root biomass with negative PC coefficients for RDW_T_ and RDW_D,_ high SDW, TN, and WUE and with stomatal limitation (PC coefficients for SC and ET were −0.265 and −0.283, respectively).

**FIGURE 3:**
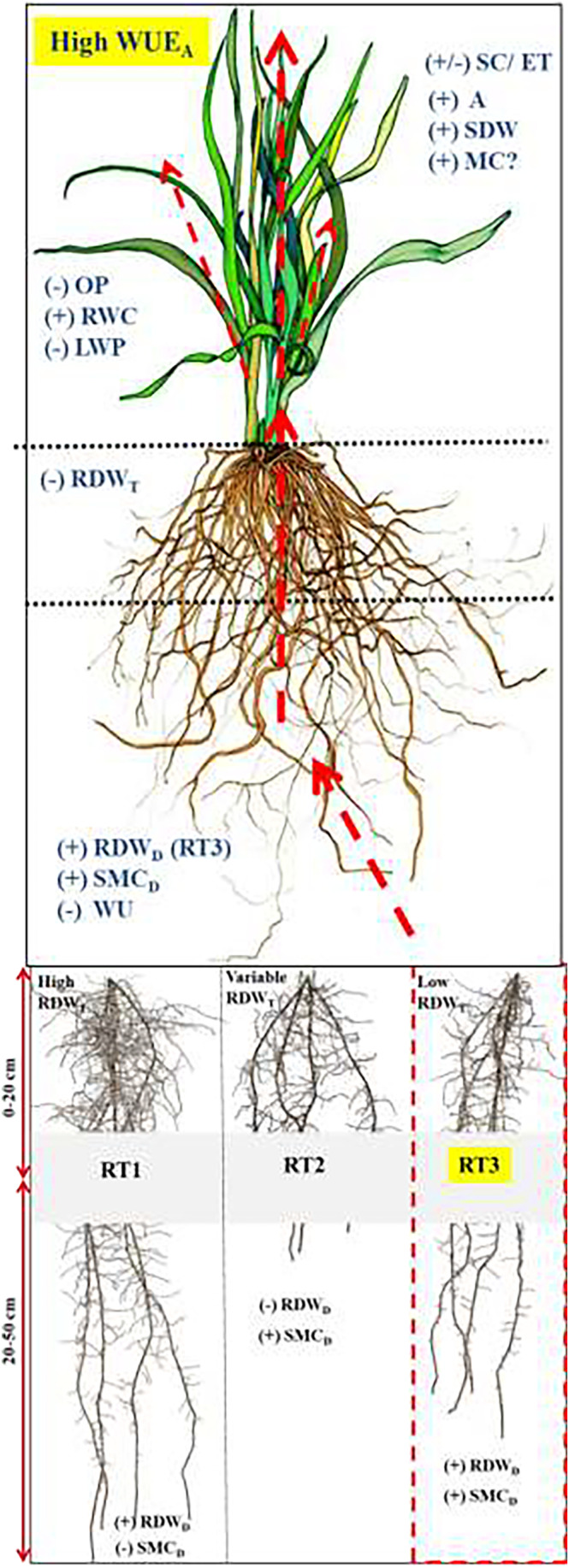
Locating key traits of a model perennial ryegrass plant for high agronomic water‐use efficiency (WUE_A_) based on the ecophysiological signature of drought response in the current results. Beginning with below‐ground traits, the proposed ideal plant has the third root type in the figure (RT3) involving enhanced RDW_D_ (deep rooting) and physiological traits that reduce SMC depletion, but reduced investment in RDW_T_. Leaf water relations are defined by more negative OP and sustained “A” leading to improved SDW in the tested moisture deficit conditions. It is postulated that enhanced MC may be involved in the trait associations expressed by the proposed ideal plant. Red arrows denote water flow in the soil plant system. A, photosynthesis (μmol m^−2^ s^−1^); ET, evapotranspiration (mmol m^−2^ s^−1^); LWP, predawn leaf water potential (MPa); MC, mesophyll conductance; OP, leaf osmotic potential (MPa); RDW_D_, root dry weight at 20–50 cm (g plant^−1^); RDW_T_, root dry weight at 0–20 cm depth (g plant^−1^); RT, root type (i.e., RT1, deep & dense [high RDW_D_ and RDW_T_]; RT2, shallow [low RDW_D_ and variable RDW_T_]; RT3, deep & sparse [high RDW_D_ and low RDW_T_] linking to PC2, PC3, and PC1, respectively, of PCA1 and accordingly, RT3 lead to an ideal soil moisture extraction strategy; see Table 5); RWC, leaf relative water content(%); SC, stomatal conductance (mmol m^−2^ s^−1^); SDW, shoot dry weight (g plant^−1^); SMC_D_, gravimetric soil moisture content at 30–40 cm depth following postcutting regrowth (%, w/w); WU, plant water use.

Taking trait associations of PC1 collectively as a mathematical description of the major drought‐response mechanism in this PRG population, Figure 3 visualizes plant characteristics including the proposed ideal root type (RT3) that features enhanced RDW_D_ and related physiological traits that reduce soil moisture depletion, but reduced investment in upper root dry weight as co‐occurring in plants with high scores for PC1 of PCA1.

### 3.3 Efficacy of alternative measures of WUE in defining efficient water use of PRG at the whole‐plant level

At MS2, Pearson correlation coefficients between the four WUE measures WUE_A_, WUE_i_, WUE_AET_, and WUE_ASC_ were statistically significant at *p* < .05 (Table 4) although they were often weak (<0.25 in four cases out of 6) and were nonsignificant (*p* > .05) at MS1 (data not shown). The highest correlation was between WUE_AET_−WUE_ASC_ (0.54; *p* < .05) followed by WUE_A_−WUE_i_ (−0.47; *p* < .05). WUE_i_ was moderately correlated with SC and RWC and highly correlated with SDW (and TN), A, and FASW at *p* < .05, while WUE_AET_ and/ or WUE_ASC_ were significantly but weakly correlated with FASW, RWC, SDW, and OP at *p* < .05 (Table 4). However, at both MS2 and MS3, WUE_A_ was significantly and highly correlated with most traits (*p* < .05; Table 4).

In PCA2, the first five PCs explaining 82.9% of data variation (eigenvalue >1) from the total of 17 PCs of all the traits measured at MS2 were selected (Table 6). PC1 of PCA2 did not retain the definition of a drought tolerance trait association linked to more negative OP, deep rootedness, and conservation of SMC_D_ as in PC1 of PCA1; however, with the omission of SMC variables and the inclusion of the four WUE measures, it resolved the WUE_A_‐WUE_i_ relationship into three independent components. PC1 showed the equivalence of the four WUE measures as indicated by comparatively large positive coefficients in each case (PC coefficients of 0.283, 0.259, 0.241 and 0.262 for WUE_A_, WUE_i_, WUE_ASC,_ and WUE_AET,_ respectively) while explaining 48% of total data variation. However, in common with PC1 of PCA1, PC1 of PCA2 also associated increased SDW with enhanced WUE but with a negative sign for FASW (Table 6). PC2 primarily identified an association between more negative OP and high RDW_D_ but also revealed a modest association between more negative OP and decreased WUE_A_ (i.e., OP and WUE_A_ coefficients had opposite signs). PC3 identified counterintuitive negative relationships between WUE_A_ and its proxy measures. With only 7% and 6%, respectively, of the total data variation explained, PC4 represented two noticeably associated traits among others (LWP and CN with PC coefficients of −0.644 and 0.465, respectively) with positive WUE_i_, WUE_ASC_, and WUE_AET_ and negative WUE_A_. PC5 identified one trait (RDW_T_, PC coefficient of −0.852) largely independent from all WUE measures (Table 6).

### 3.4 Quantitative genetics of drought tolerance‐related morpho‐physiological traits, WUE_A_ , and proxy measures of WUE_A_ in PRG

Table 3 shows the quantitative genetic parameters for MS2 and MS3, but not for MS1 because the genetic variation was nonsignificant (*p* > .05) for most traits in early drought (MS1). For the selected subset of half‐sib families, most plant traits exhibited higher genetic among‐half‐sib family ($\sigma_{f}^{2}$) and within‐half‐sib family ($\sigma_{f/s}^{2}$) variances (excluding SC) and narrow‐sense heritability ($h_{n}^{2}$) estimates at MS3 than at MS2 (*p* < .05; Tables 7 and 8, respectively). For most traits measured at MS2, the $h_{n}^{2}$ ranged from 0.57 to 0.82 (excluding nonheritable RDW_T_ and gas exchange traits; Table 7) while the $h_{n}^{2}$ of the same set of traits ranged from 0.84 to 0.96 at MS3 (Table 8). For example, $h_{n}^{2}$ estimates of WUE_A_ at MS2 and MS3 were 0.61 and 0.92, respectively. For WUEi, $h_{n}^{2}$ was above 0.75 at MS2 but 0.42 at MS1 (WUE_i_ was not measured at MS3 as explained elsewhere). By contrast, $h_{n}^{2}$ estimates for WUE_AET_ and WUE_ASC_ were statistically nonsignificant at both MS2 and MS3 (*p* > .05; Tables 7 and 8) but statistically significant at MS1 (i.e., $h_{n}^{2}$ of WUE_AET_ and WUE_ASC_ were 0.78 and 0.47, respectively, at *p* < .05). The coefficient of variation observed for the heritable traits was within an acceptable range for plant breeding purposes (i.e., below 30%) at both MS2 and MS3 (Tables 7 and 8).

Predicted genetic gain (ΔG_c_) estimates of most traits were higher at MS3 than at MS2 (Table 9). The highest ΔG_c_ estimates of among‐family‐selection (AFS) and among‐and‐within‐family‐selection (AWFS) selection at 30% of selection pressure were for RDW_D_ (9%–18%) followed by RGS and WUE_A_ (4%–8%) among all traits of interest. At MS2, ΔG_c_ estimates of AFS and AWFS for WUEi ranged from 2% to 4% (Table 9), similar to that observed at MS1 (data not shown). However, ΔG_c_ estimates of WUE_AET_ and WUE_ASC_ were less than those of WUE_A_ or WUE_i_ at both MS2 and MS3. Gas exchange measurements (i.e., A, SC, and ET) showed more significant ΔG_c_ estimates at MS1 than at MS2 and MS3 (data not shown).

Despite the large variations in the phenotypic correlation coefficients of key traits within the subset of half‐sib families (i.e., 0.1–0.9; *p* < .05; see Table 4 and Table S1a and S1b), *r*_g_ coefficients estimated for the heritable trait pairs showed a narrow range (i.e., 0.6–0.9) at both MS2 and MS3 (data not shown). However, there were a few exceptions for WUE_A_‐OP, WUE_A_‐RDW_D_, WUE_A_‐SC, OP‐RDW_D_, and OP‐SC trait pairs, where their *r*_g_ coefficients ranged from 0.3 to 0.5 in both MS2 and MS3 (*p* < .05; data not shown). Correlated response to selection estimates of highly genetically correlated traits was generally similar (e.g., WUE_A_‐RWC and WUE_A_‐A trait pairs while WUE_A_ was the primary trait) or mostly lower (e.g., WUE_A_‐OP, WUE_A_‐RDW_D_, and WUE_A_‐SC while WUE_A_ was the primary trait) compared with the ΔG_c_ of single‐trait selection estimated for each key trait (data not shown). ΔG_c_ by AFS and AWFS of WUE_i_ and WUE_A_ ranged from 2% to 4% and from 4% to 8%, respectively, (see above) while correlated response to selection estimate of WUE_A_‐WUE_i_ ranged from 2% to 4% at the 30% selection pressure (*p* < .05). However, the correlated response to selection estimate of the OP‐RDW_D_ trait pair (10%–20%) was larger than the ΔG_c_ estimates of OP (3%–6%) and RDW_D_ (9%–18%; Table 9).

## 4 DISCUSSION

### 4.1 Morpho‐physiological trait associations defined a key PRG dehydration avoidance mechanism

Osmotic adjustment, which is the active lowering of solute potential due to the accumulation of solutes in plant cells at low predawn leaf water potential (LWP), is one of the most common drivers of plant dehydration avoidance and is a pathway for maintaining both the production (Cyriac et al., 2018; Turner, 2018) and survival (Blum, 1989, 2017; Blum & Tuberosa, 2018; Turner, 2018) of crop plants under prolonged drought. Scientists typically refer to osmotic adjustment as osmoregulation, which is defined as the addition of osmolytes to the cell sap during the drought response (Turner, 2018) and is thus distinguished from more negative OP which is induced by falling RWC as cell solutes become more concentrated (Cyriac et al., 2018). Notably, our results show that (1) the OP decrease of some genotypes was derived from the increase in cell sap concentration with declining RWC whereas (2) another large group of genotypes indicated signs of active solute accumulation (or addition) in the cell sap (see Figure 2d above) corresponding to the trait association explained by PC1 of PCA1 (see Table 5). Moreover, PRG plants of (2) above had higher RWC values at more negative LWP and more negative adjusted OP levels at MS3 than at MS2 (Figure 2). This may indicate that those PRG plants have the capacity to repair stress‐induced damage while maintaining the integrity of physiological functions, even under intense drought (see below).

Our results challenge the view that osmotic adjustment is merely related to drought survival through enhanced rooting and prolonged soil moisture capture at the expense of crop yield (Kang et al., 2022). The trait association explained by PC1 of PCA1 (Table 5) included high yield and retention of net assimilation capacity during drought by a set of inter‐related traits as supported by improved leaf hydration with signs of osmotic adjustment (i.e., correlation between OP_100_ (MS3)–OP_100_ (MS2) and PC1 scores of PCA1 was 0.53; data not shown) and reduced soil moisture depletion (positive SMC_D_) or regulated plant water use (high WUE_A_ or low WU). These trait expressions together seemed to constitute a constructive dehydration avoidance mechanism (hereafter referred as WUE_A_‐OP mechanism) of PRG. This trait expression is however inconsistent with the EUW concept advocated by Blum (2009) as defined elsewhere, because EUW would imply that deep rooting enhances yield through increased water uptake rather than through SMC preservation for subsequent use which is obviously unnecessary for annual crops but advantageous for perennial plants including PRG. In this respect, our results highlight the importance of the deep and sparse rooting pattern in some PRG plants (i.e., Root type 3 [PC1 of PCA1]; high RDW_D_ and low RDW_T_ linked to well‐regulated SMC extraction) over deep and dense rooting features and indicate balanced plant dry matter partitioning features in the WUE_A_‐OP trait mechanism of drought‐tolerant PRG (i.e., Root type 1 [PC2 of PCA1]; high RDW_T_ and RDW_D_ linked to excessive SMC uptake) or shallow‐rooting PRG (i.e., Root type 2 [PC3 of PCA1]; variable RDW_T_ and low RDW_D_ linked to needless SMC conservation). Zhan et al. (2015) and Ruggiero et al. (2017) considered that growth and expansion of the lateral root system beyond the minimal requirements for water uptake for net assimilation can come at a cost to the plant's carbon budget under limited water availability. Such a “cost” is consistent with our observation of a link between restricted RDW_T_ features and the expression of other key water relations traits in PC1 of PCA1. This pattern identified by PC1 in which soil moisture retention occurs simultaneously with traits related to water extraction and ongoing growth has seldom if ever been reported previously. Stabilization of this trait association in a PRG breeding population would represent a major advance in PRG breeding for drought tolerance.

### 4.2 WUE_A_ was the most informative WUE measure in defining PRG drought tolerance

Considering the practical difficulties of measuring WUE_A_ precisely in field settings and the apparent physiological linkage between WUE_A_ and δ^13^C, WUEi is often used as an easily measurable proxy measure of WUE_A_ in selecting plant genotypes for improved WUE and drought tolerance despite inadequate scientific evidence up to date (Avramova et al., 2019; Condon, 2020; Farquhar & Richards, 1984). Thus, through simultaneous measurements for the same plants under drought, our results confirmed that WUE proxy measures are not identical measures of WUE_A_ unless resolved into their mutually inclusive component mechanisms or trait associations. Only then can δ^13^C‐based WUE determinations fully support efficient selection of PRG for drought tolerance from a plant physiology perspective.

PC1 of PCA2 explaining 48% of data variation (Table 6) captured the theoretically predicted positive correlation between WUE_A_, WUE_i_, WUE_ASC_, and WUE_AET_ and indicated that numerically high WUE (i.e., low WUE or more WU per g DW produced) is linked to low SDW and TN (i.e., negative coefficients for SDW and TN) and restricted net assimilation (i.e., negative coefficients for A, SC, and ET). Interestingly, the reduced SDW scenario linked to low WUE results in increased retention of available soil water (coefficient for FASW was +0.313), and OP was not involved in this trait association. This dehydration tolerance scenario is consistent with literature where reduced SDW under water deficit was associated with reduced soil moisture depletion and vice versa (Blum, 2005; Blum & Tuberosa, 2018). Conversely, the reverse dehydration avoidance scenario indicated by the opposite tail of PC1 scores or reversing PC1 signs in Table 5 (i.e., increased SDW was accompanied by increased soil moisture depletion) was partially consistent with Blum's (2009) EUW concept but differs from it in that WUE_A_ was actually numerically reduced alongside the reduction of SDW. This indicates that improved WUE with high SDW may in return trigger a yield reduction with restricted WU in drought that Blum (2005) was concerned about. The PCA2‐PC1 scenario also differs critically from the trait association revealed by PC1 of PCA1, in that increased SDW is linked to increased (not reduced) soil moisture depletion. The different plant physiology insights coming from the two PCAs likely reflect the different trait data included in each PCA.

With respect to agreement between WUE_A_ data and the three proxy measures, by definition, instantaneous WUE (i.e., WUE_ASC_ or WUE_AET_) measurements of photosynthetic gas exchange derived from data collected at one or a few time points may not completely represent the mean growth conditions over the entire drought cycle that determine WUE for harvested biomass (Flexas et al., 2016; Seibt et al., 2008). By contrast, WUE_A_ data and the plant δ^13^C signal of WUE_i_ capture long‐term effects of stomatal limitations of net assimilation under drought but are influenced by other operative morpho‐physiological responses including mesophyll conductance, carboxylation, C:N allocation, and leaf structure (Condon, 2020; Seibt et al., 2008). PCs 2, 3, 4, and 5 of PCA2 reflect other trait associations that are likely related to particular physiological processes such as those just mentioned and are by definition mathematically uncorrelated with the pattern in PC1. Such effects likely obscure the underlying fundamental relationships among the four measures of WUE in real‐world data. For example, PC2 indicates that, when PCA2 was corrected for the effects of other traits, more negative OP acted to modestly decrease WUE_A_ (i.e., OP and WUE_A_ coefficients have opposite signs) and had near‐zero influence on gas exchange parameters or WUE_AET_ and WUE_ASC_ (i.e., A, SC, ET, WUE_AET,_ and WUE_ASC_ coefficients all had absolute value <0.095), although there was a strong association between more negative OP and increased RDW_D_. This is counterintuitive but discounts the possibility that the physiological mechanism of the WUE_A_‐OP trait association might be a conserving effect of more negative OP on A and ET.

In our results, PC3 of PCA2 indicated an uncoupling between the integrative measures of WUE (WUE_A_ and WUE_i_) and the instantaneous measures of WUE_ASC_ and WUE_AET_ (i.e., PC coefficients of integrative and instantaneous have opposite signs) with WUE_ASC_ and WUE_AET_ numerically high (indicating low WUE) when ET and SC were high (Table 6). This uncoupling of WUE_A_ data and proxy measures based on gas exchange data could happen if gas exchange data were collected when conditions differed from the “average” conditions prevailing during the longer timeframe of WUE_A_ data collection. This provides an objective evaluation of the extent to which instantaneous WUE data from gas exchange measurements were affected by this issue in this experiment. Both the physiological realities captured in PC2 and PC3 and the independence of fundamental WUE expressed in PC1 of PCA2 would contribute data noise obscuring the correlation between WUE_A_ and its proxy measures and that may explain why proxy measures are inefficient predictors in some experiments.

A point for future research is the role of genotypic variation in mesophyll conductance in the WUE_A_‐OP trait association detected in PC1 of PCA1. Mesophyll conductance is a critical variable for the use of δ^13^C to infer photosynthetic WUEi (Condon, 2020; Olsovska et al., 2016; Stangl et al., 2019) and thereby overall plant WUE_A_. Moreover, co‐regulation or mutual independence between SC and mesophyll conductance may contribute to the photosynthetic response under increasing stomatal limitation under intense or prolonged drought conditions (Condon, 2020; Olsovska et al., 2016). For example, Stangl et al. (2019) described an asynchronous diurnal behavior of mesophyll conductance and SC of *Pinus sylvestris* where SC declined from around 10.00 am, A started declining after midday, and mesophyll conductance remained near its maximum until 4.00 pm, suggesting that high mesophyll conductance plays a role in supporting extended A, despite stomatal limitation in drought. Therefore, further research should determine whether mesophyll conductance contributes to the improved yield with restricted SMC uptake as per the WUE_A_‐OP trait mechanism, irrespective of the possible stomatal limitations below 65% FC (see Figure 3).

### 4.3 Estimates of quantitative genetic parameters reinforced the importance of the WUE_A_‐OP trait mechanism and the direct measure of WUE_A_ over its proxy measures for deducing PRG drought tolerance under soil drought

Our results highlighted the integrated trait response of PRG drought tolerance through improved WUE_A_, more negative OP, deep rootedness, and SMC conservation, conferring a yield advantage under imposed drought. Our findings also demonstrated a partial agreement between easily measurable WUE_i_ and WUE_A_ data (see above). However, before using any of the trait/s of interest for selection and pyramiding of correlated traits in drought‐tolerant PRG genotypes, information on the key quantitative genetics on both individual (i.e., direct response to selection; ΔG_c_) and correlated (i.e., correlated response to selection) trait responses is required. In selection programs, heritability is the primary parameter that numerically quantifies whether the progress from selection for a given trait (s) will be straightforward or challenging (Schmidt et al., 2019). In particular, narrow‐sense heritability ($h_{n}^{2})$ describes the ratio of additive genetic variance, which allows permanent and continued response to selection, to phenotypic variance of measured traits (Schmidt et al., 2019).

In our results, $h_{n}^{2}$ of most drought tolerance traits was >0.5, indicating that more than half of the differences among phenotypes of the traits of interest were genetically determined under the conditions tested. Moreover, WUE_A_ showed >60% (including WUE_i_) and 90% genetic potential at MS2 and MS3, respectively (see Tables 7 and 8), which may be indicative of an agronomically useful response to selection for those traits. Supporting this, our study that laid the foundation for the present work (see Weerarathne et al., 2021) revealed that conventional pasture breeding has indirectly resulted in high WUE_A_ and drought tolerance in modern PRG cultivars. This was further confirmed in other related studies (Chapter 3 of Weerarathne, 2021; Weerarathne et al., 2018). As expected, calculated ΔG_c_ or the estimate of prediction of the expected response to selection was high for WUE_A_ and ranged from moderate to high for related traits including WUE_i_ at MS2 and MS3 (see Table 9). Thus, the results suggest that WUE_A_ and other traits of interest (i.e., SDW including TN and RGS, RDW_D_, and OP; see Table 9) are strong candidates for progression of their genetic potential through selection.

In practice, laborious trait measurements including WUE_A_ may benefit from indirect selection when the most appropriate secondary trait (i.e., WUE_i_; see above) is cheaper and/or easier to measure in larger populations and is under higher selection pressure than that of the primary trait (Babar et al., 2007). This alternative approach is usually considered if r_g_ between two target traits is very high and $h_{n}^{2}$ is higher for the secondary target trait than for the primary trait, and also, if correlated response to selection estimate of both traits is considerably higher than ΔG_c_ of the secondary trait (Babar et al., 2007). However, with modest phenotypic and genetic correlations observed between WUE_A_ and WUE_i_ regardless of the higher $h_{n}^{2}$ estimate of WUE_i_ than that of WUE_A_ at MS2 (i.e., 0.76 and 0.61, respectively; Table 7), correlated response to selection estimates of the indirect selection of PRG genotypes for WUE_i_ was just similar to its ΔG_c_ value. (while lower to that of WUE_A_) under the conditions tested. Therefore, from both physiological and quantitative genetics perspectives, our results suggest that it is advantageous to select drought‐tolerant PRG plants directly based on WUE_A_ (preferably late during drought where the highest genetic potential is expressed; see above) than indirectly based on WUE_i_. Besides, the correlated response to selection estimates of highly genetically correlated and heritable key traits with WUE_A_ (i.e., OP, RDW_D_, RGS, and A) was lower or similar to ΔG_c_ of each secondary trait, indicating that WUE_A_ should be supported with such traits to achieve genetic gain in PRG drought tolerance through selection. Interestingly, correlated response to selection estimate of OP and RDW_D_ was higher than the ΔG_c_ estimates of both traits, suggesting that one trait would be adequate (with or without the backup trait; SMC_D_) as a joint selection criterion alongside WUE_A_ to improve PRG drought tolerance.

In general, prolonged drought causes a significant decrease in A under conditions when the stomata of most cultivated plants begin to close and when LWP goes below −1.3 MP as to reduce ET (Jones et al., 1980; Xie et al., 2020). However, in the PCA results (explaining 56% of phenotypic data variation), gas exchange measures showed potential as another selection criterion for PRG drought tolerance alongside traits related to the WUE_A_‐OP mechanism even at lower LWP levels (i.e., average LWP values were −1.1 and −1.4 MPa, respectively, at MS2 and MS3). Supporting this, A showed genetic potential with high $h_{n}^{2}$ and significant *r*_g_ estimates with SC, ET, OP, WUE_A_, and WUE_i_ traits at both MS2 and MS3. Nevertheless, $h_{n}^{2}$ estimates of WUE_AET_ and WUE_ASC_ were statistically nonsignificant for the data measured at both MS2 and MS3, indicating their nonheritable nature under imposed drought. This may be partially because the $h_{n}^{2}$ of SC was significant but additive genetic variance was statistically nonsignificant especially at MS3 (see Table 8), implying its additive‐by‐additive epistasis nature in the chosen half‐sib family plants. According to Cheverud and Routman (1996), with additive‐by‐additive epistasis, genetic variance at each locus that makes the trait of interest heritable is additive, but the expression of additive effects may vary or be absent depending on the frequency of alleles of each locus from generation to generation. Further research on the quantitative genetics of nonstomatal limitations and their impact on photosynthetic WUE is warranted to clarify this genetic response.

In summary, our results favored selecting PRG for WUE_A_ over its proxy measures because it is the key to the WUE_A_‐OP trait association, together with its higher genetic potential and repeatability (followed by WUE_i_) compared with those of WUE_ASC_ and WUE_AET_ under the conditions tested. Our results further showed that selecting PRG plants for certain component traits of the WUE_A_‐OP trait mechanism would be advantageous in sustaining SMC retention for drought tolerance. Notably, OP or RDW_D_ can be excluded from the selected trait combination as their r_g_ and correlated response to selection estimates were exceptionally high and thus selecting PRG genotypes for one trait will naturally bring in the other. In previous research, $h_{n}^{2}$ and ∆G_c_ estimates of OP of PRG were found to be significantly high and consistent (Thomas, 1990) while we detected the highest ∆G_c_ for RDW_D_ (see Table 9). Moreover, from a plant physiology perspective, more negative osmotic potential may lead to both SMC extraction (see PC2 of PCA1, Table 5; RT1, Figure 3) and SMC conservation (see PC1 of PCA1, Table 5; RT3, Figure 3) of high‐WUE_A_ plants. Thus, PRG plants should simultaneously be screened for both more negative leaf OP and SMC at depth in the absence of RDW data in order to precisely identify the genotypes with the most desirable trait associations for drought tolerance. Besides, further studies on the trait combinations that strengthen the interconnection between WUE_A_ and WUE_i_ would potentially benefit PRG improvement because WUE_i_ also allows high‐throughput phenotyping for WUE_A._ Nonetheless, based on data reported here, WUE_i_ represents nearly half of the genetic variability of WUE_A_ related to PRG drought tolerance. As the next steps, field validation of performance of plants selected for key traits identified in this study (including WUE_A_ [and WUE_i_] and RDW_D_ or OP [with SMC_D_] and also, A), investigation of the potential role of mesophyll conductance in the WUE_A_‐OP trait association, characterization of the QTLs of WUE_A,_ and evaluation of the relevance of these results to forage species other than PRG are highly desirable.

## CONFLICT OF INTEREST STATEMENT

No potential conflict of interest was reported by the authors.

## Supporting information


Appendix S1.

Appendix S2.

Appendix S3.

## Data Availability

The original dataset that supports the findings of this study is openly available in Appendix S3.
