# Supplementary material for: A comparative analysis of agronomic water‐use efficiency and its proxy measures as derived from key morpho‐physiological and supportive quantitative genetics attributes of perennial ryegrass under imposed drought
Source: Plant Environ Interact. 2023 Aug 30;4(5):291–307. doi: 10.1002/pei3.10123 (PMC10565840; doi:10.1002/pei3.10123)
Supplement: Supplementary file 1 — Appendix S1. [file PEI3-4-291-s001.docx]

***SI-1 Detailed Methods of Quantitative Genetic Analyses***

The Residual Maximum Likelihood (REML) procedure, based on a complete random linear model (Eq.1), was applied to generate Best Linear Unbiased Predictor (BLUP) values (White & Hodge, 2013) using the software DeltaGen (v. 0.03) (Jahufer & Luo, 2018).

$Y_{ijklmn}=M+ f_{i}+s_{ij}+b_{k}+{(fb)}_{ik}+r_{kl}+c_{km}+\varepsilon_{ijklmn}$ (Eq.1)

Where, Y_ijklmn_ is the value of an attribute measured from sample j in HS family i in row l and column m of replicate k and i=1,...,n_f_, j=1,…,n_s_, k=1,...,n_b_, l=1,...,n_r_, m=1,...,n_c_, where f, s, b, r and c are HS families, samples, replicates, rows and columns, respectively; M is the overall mean; f_i_ is the random effect of HS family i, N(0,σ^2^_f_); s_ij_ is the random effect of sample j in family i, N(0,σ^2^_f/s_); b_k_ is the random effect of replicate k, N(0,σ^2^_b_); (fb)_ik_ is the random effect of the interaction between family i and replicate k, N(0, σ^2^_fb_); r_kl_ is the random effect of row l within replicate k, N(0,σ^2^_r_); c_km_ is the random effect of column m within replicate k, N(0,σ^2^_c_); ε_ijklmn_ is the residual effect of sample j in HS family i in row l and column m of replicate k, N(0,σ^2^_ε_).

The narrow-sense heritability $h_{n}^{2}$ of each trait was estimated on half-sib family mean basis across replicates using DeltaGen software, based on Eq.2;

$h_{n}^{2}=\frac{\sigma_{A}^{2}}{\sigma_{A}^{2}+\frac{\sigma_{Ab}^{2}}{n_{b}}+\frac{\sigma_{\varepsilon}^{2}}{n_{s}n_{b}}}$ (Eq.2)

Where, $\sigma_{A}^{2}$; additive genetic variance (1/4 $\sigma_{A}^{2}$ as estimated among half-sib families), $\sigma_{\mathrm{Ab}}^{2}$, additive-by-replicate interaction and $\sigma_{\varepsilon}^{2}$, experimental error; $n_{b}$, number of replicates, $n_{s}$, number of samples.

Predicted genetic gain per cycle of selection (ΔG_c_), based on only among half-sib family selection (Eq. 3a) and also the combination of among and within (Eq. 3b) half-sib family selection (Casler and Brummer, 2008) was estimated using DeltaGen;

${\Delta G}_{\mathrm{HSF}}=k_{f}c\frac{{\frac{1}{4}\sigma}_{A}^{2}}{\sigma_{\mathrm{PF}}}$ (Eq.3a)

Where, ${\Delta G}_{HSF}$, predicted genetic gain using only among half-sib family selection; $\sigma_{A}^{2}$, additive genetic variance; $\sigma_{\mathrm{PF}}$, among half-sib family phenotypic standard deviation; $k_{f}$, among HS family selection intensity; $c$, parental control (0.5).

${\Delta G}_{AWF-HS}=k_{f}c\frac{\frac{1}{4}\sigma_{A}^{2}}{\sigma_{\mathrm{PF}}}+k_{\mathrm{wf}}c\frac{\frac{3}{4}\sigma_{A}^{2}}{\sigma_{\mathrm{PWF}}}$ (Eq. 3b)

Where, ${\Delta G}_{AWF-HS}$, predicted genetic gain using the combination of among and within half-sib family selection; $\sigma_{A}^{2}$, additive genetic variance; $\sigma_{\mathrm{PF}}$, among half-sib family phenotypic standard deviation; $\sigma_{\mathrm{PwF}}$, within half-sib family phenotypic standard deviation; $k_{f}$, among HS family selection intensity;$k_{\mathrm{wf}}$, within HS family selection intensity; $c$, parental control (0.5).

The multivariate analysis of variance option in DeltaGen software that generates variance and covariance matrices was used to estimate genetic correlation (r_A_) between trait pairs of the key drought-response trait association/ s (Eq.4);

$r_{A}=\frac{\mathrm{COV}_{\mathrm{XY}}}{\surd\sigma_{A_{x}}^{2}\sigma_{A_{y}}^{2}}$ (Eq.4)

Where $\mathrm{COV}_{\mathrm{XY}}$, additive genetic covariance between traits X and Y, $\sigma_{A_{x}}^{2}$, the additive genetic variance of trait X and, $\sigma_{A_{y}}^{2}$, additive genetic variance of trait Y.

The correlated response to selection (*CRy*) of the primary trait *Y*, when selection was applied to a secondary trait *X*, at 30% of selection pressure, was predicted using the following equation (Eq.5) in DeltaGen;

${CR}_{y}=i_{x}h_{x}h_{y}r_{A}\surd V_{py}$ (Eq.5)

Where i_x_ is the selection intensity of the secondary trait, h_x_ and h_y_ are square roots of the heritability of the traits *X* and *Y*, respectively, r_A_ is the genetic correlation between traits, and $\sqrt{V_{\mathrm{py}}}$ is the square root of the phenotypic variance of the primary trait (Acquaah, 2015).
