# Supplementary material for: A comparative analysis of agronomic water‐use efficiency and its proxy measures as derived from key morpho‐physiological and supportive quantitative genetics attributes of perennial ryegrass under imposed drought
Source: Plant Environ Interact. 2023 Aug 30;4(5):291–307. doi: 10.1002/pei3.10123 (PMC10565840; doi:10.1002/pei3.10123)
Supplement: Supplementary file 2 — Appendix S2. [file PEI3-4-291-s002.docx]

**Table S1a** Pearson correlation coefficients of the plant water relations (TA1) and morpho-physiological (TA2) traits of perennial ryegrass half-sib family population evaluated within and between the measurement stages 1 (field capacity; FC), 2 (55–65% FC), and 3 (45–55% FC). Only significant correlation coefficients are presented.

*p<0.05; **p<0.001 (Trait abbreviations: see below).

**Table S1b** Pearson correlation coefficients between key traits of the plant-soil-water relations (TA1) and morpho-physiological (TA2) groups as classified in Table 3a above at the three consecutive measurement stages (1,2, and 3) of the current experiment. Only significant correlation coefficients are presented.

*p<0.05; **p<0.001 (Trait abbreviations: see below).

**
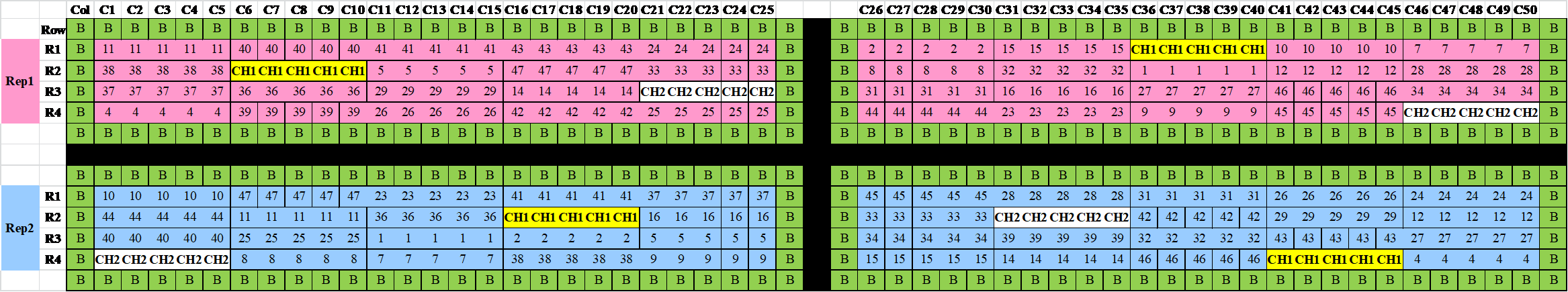
**

**Figure S1** Row–column experimental design with repeated checks (CH)

(Note: Two replicates of five genotypes from 36 representative perennial ryegrass half–sib families of an advanced breeding population were included with 40 check plants in this experiment. The border plants are indicated as B).

**Trait Abbreviations:** SDW (g plant^–1^); Shoot dry weight, C:N; Carbon: Nitrogen ratio, RDW_T_ (g plant^–1^); Top root dry weight (0–20 cm), RDW_D_ (g plant^–1^); Deeper root dry weight (20–50 cm), RGS; Post-cutting regrowth score, TN; Tiller number, A (µmol m^–2^ s^–1^); Photosynthesis, SC (mmol m^–2^ s^–1^); Stomatal conductance, ET (mmol m^–2^ s^–1^); Evapotranspiration, Δ^13^C; Carbon isotope discrimination of oven-dried (1h at 105ºC and 48h at 60ºC) fully expanded youngest leaf samples from representative tillers, RWC (%); Leaf relative water content, OP (MPa); Leaf osmotic potential, LWP (MPa); Predawn leaf water potential, SMC_T_ (%, w/w); Gravimetric soil moisture content at top soil layers (10–20 cm), SMC_D_ (%, w/w); Gravimetric soil moisture content at deeper soil layers (40–50 cm), FASW (%, w/w); Remaining fraction of soil available water, WUE_A_ (g H_2_O g^–1^ DW); Agronomic water-use efficiency, WUE_ASC_ and WUE_AET_; Instantaneous WUE (gas exchange-based; µmol mol^-1^), WUEi; Intrinsic WUE (Δ^13^C-based; µmol mol^-1^).
